# Effective amelioration of the Lucio phenomenon with adjuvant tofacitinib therapy in a patient with dual infection of *Mycobacterium leprae* and *Mycobacterium lepromatosis*: a case report from India

**DOI:** 10.1099/acmi.0.000460.v3

**Published:** 2023-10-20

**Authors:** Nayankumar Harshadkumar Patel, Jignaben Krunal Padhiyar, Kathak Ashok Patel, Jahnvi Rakeshkumar Patel, Megha Pratapabhai Lakum, Itu Singh

**Affiliations:** ^1^​ GCS Medical College Hospital and Research Centre, Ahmedabad, India; ^2^​ Stanley Browne Laboratory, TLM Community Hospital, Nand Nagari, Delhi, India

**Keywords:** dual infection, lepra lymphadenitis, *Mycobacterium leprae*, *Mycobacterium lepromatosis*, tofacitinib

## Abstract

**Introduction.:**

The Lucio phenomenon (LP) is a characteristic reaction pattern seen in patients with diffuse lepromatous leprosy (DLL). Dual infection with *Mycobacterium leprae* and *Mycobacterium lepromatosis* in DLL has been confirmed from other endemic countries but not previously documented from India. Conventionally, LP is treated with a high dose of systemic glucocorticoid (GC) and anti-leprosy treatment (ALT). Here we report a case of leprosy lymphadenitis at initial presentation in a patient with LP and DLL due to dual infection with *M. leprae* and *M. lepromatosis* who responded favourably to tofacitinib as adjuvant to ALT and systemic GC therapy.

**Case report.:**

A 20- to 30-year-old man presented with swelling over the bilateral inguinal region, pus-filled skin lesions with multiple ulcers, fever and joint pain. Post-hospitalization investigations showed the presence of anaemia, leukocytosis, and elevated acute and chronic inflammatory markers. Skin and lymph node biopsies were suggestive of LP and leprosy lymphadenitis. The presence of *M. leprae* and *M. lepromatosis* was confirmed by PCR followed by DNA sequencing of PCR amplicons from tissue. Despite anti-leprosy treatment, oral GC and thalidomide therapy, the patient continued to develop new lesions. One month after the commencement of adjuvant tofacitinib, the patient showed excellent clinical improvement with healing of all existing lesions and cessation of new LP lesions.

**Conclusion.:**

Our case confirms the presence of dual infection with *M. leprae* and *lepromatosis* in India. Lymph node involvement as an initial presentation of DLL should be considered in endemic areas. Tofacitinib may be a promising new adjuvant therapy for recalcitrant lepra reactions.

## Data Summary

All data related to this case detail are given in this report. No new data were generated over and above this.

## Case report

### Introduction

The Lucio phenomenon (LP) is a characteristic reaction pattern seen in patients with diffuse lepromatous leprosy (DLL) characterized by ulceronecrotic lesions. Infection with *Mycobacterium lepromatosis* has been reported from many endemic countries as a cause of DLL and LP. Dual infection of *Mycobacterium leprae* and *M. lepromatosis* has been confirmed from other endemic countries but not previously documented from India. Though involvement of lymph nodes in leprosy and the lepra reaction is not uncommon, generalized lymphadenopathy as an initial presentation is not routine. Conventionally, LP is treated with a high dose of systemic glucocorticoid (GC) and anti-leprosy treatment (ALT). Here we report a case of leprosy lymphadenitis at initial presentation in a patient with LP and DLL due to dual infection with *M. leprae* and *M. lepromatosis* who responded favourably to tofacitinib as adjuvant to ALT and systemic GC therapy. Studies have shown that the pathogenesis of the type 2 lepra reaction involves increased Th2 cytokine expression (e.g. IL4, IL5, IL6), with recent evidence of Th17 cell involvement. Tofacitinib is a reversible inhibitor of the enzyme Janus kinase (JAK) that effectively diminishes the intracellular signal transduction of the above-mentioned cytokines.

### Case presentation

**Fig. 1.**
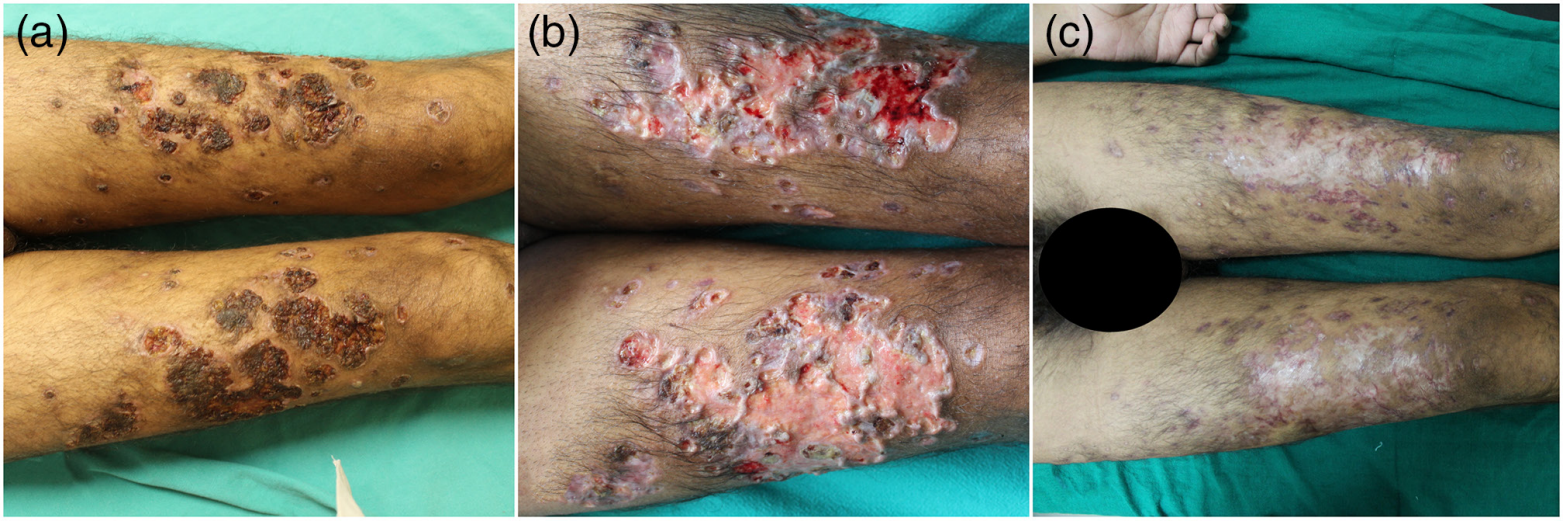
(a) Extensive ulceronecrotic lesions with haemorrhagic crusting bilaterally. Intake pustules are visible at the periphery. (b) Persistent cutaneous ulceration 2 months after starting therapy. (c) Complete healing of skin lesions 1 month after starting adjuvant tofacitinib.

A 20- to 30-year-old man presented with swelling over the bilateral inguinal region, pus-filled skin lesions with multiple ulcers, fever and joint pain. The asymptomatic inguinal swelling had been present for the last 5 months for which he was on anti-tubercular drugs. He started to develop skin lesions which were associated with fever and joint pain 2 months before his presentation. On examination the patient had multiple well-defined punched out ulcers of varying sizes with violaceous margins and necrotic haemorrhagic slough, present predominantly over both extremities (Fig. 1a).

Multiple tender and enlarged lymph nodes were present in bilateral inguinal, axillary and cervical regions (largest measuring 3×2 cm). There were no signs of ear lobe infiltration, nerve thickening or sensory loss. Initial clinical differentials of pityriasis lichenoides et varioliformis acuta, LP and pyoderma gangrenosum were considered. Post-hospitalization investigations showed the presence of anaemia, leucocytosis, and elevated acute and chronic inflammatory markers (Table 1).

**Table 1.** Sequential details of laboratory, radiological and microbiological investigations of case

Investigation	At presentation	1 month post-therapy (ALT+steroid+thalidomide)	2 months post-therapy (adjuvant adalimumab 2 dose of 40 mg, 15 days apart)	3 month post-therapy (adjuvant tofacitinib 5 mg twice day)
Haemoglobin (g dl^−1^)	8.4	9.9	8.6	10
Total leucocyte count (cells mm^–3^)	21910	16710	32310	7000
Erythrocyte sedimentation rate (mm h^–1^)	110	36	80	50
C-reactive protein (mg/L)	289.25	129.85	185.6	99.18
Ferritin (ng ml^−1^)	622.8			
d-Dimer (ng ml^−1^)	1355.4			
Microbiological investigations	No organism isolated on aerobic and anaerobic culture. Modified ZN stain shows presence of acid fast bacilli in pus. * M. tuberculosis* not detected using culture and cartridge-based amplification test. Presence of *M. leprae* was confirmed in skin tissue using PCR. *M. leprae* specific repetitive element (RLEP)-forward 5′-TGCATGTCATGGCCTTGAGG-3′ and RLEP-reverse 5′-CACCGATACCAGCGGCAGAA-3′ and product size 129 bp. Presence of *M. lepromatosis* was confirmed in skin and lymph node tissues via heminested PCR using protocol developed by Han and Quintanilla in 2015 [5]. First round of PCR was done with primers AFBO (5′-GCGTGCTTAACACATGCAAGTC, common to all mycobacterial species) and MLER4 (5′-CCACAAGACATGCGCCTTGAAG, specific for *M. leprae *); product size is 171 bp. It was diluted 100-fold and further amplified with three separate second rounds of PCR using MLER4 as the anchoring primer to pair with primer LPMF2 (5′-GTCTCTTAATACTTAAACCTATTAA, specific for *M. lepromatosis*); amplicon of 142 bp in size. It was further confirmed by DNA sequencing of PCR product, which showed 100 % similarity with *M. lepromatosis*.			
Imaging studies	Chest X-ray: no abnormality detected. Ultrasonography of lymph node: multiple enlarged lymph nodes in bilateral inguinal, axilla and cervical region. Largest measuring 25×9 mm in right inguinal region, 19×6 mm in left inguinal region, 23×11 mm in right axilla, 21×12 mm in left axilla and 20×11 mm in left level 1B. All lymph nodes show altered echotexture with internal areas of necrosis.			

ALT, anti-leprosy treatment.

**Fig. 2.**
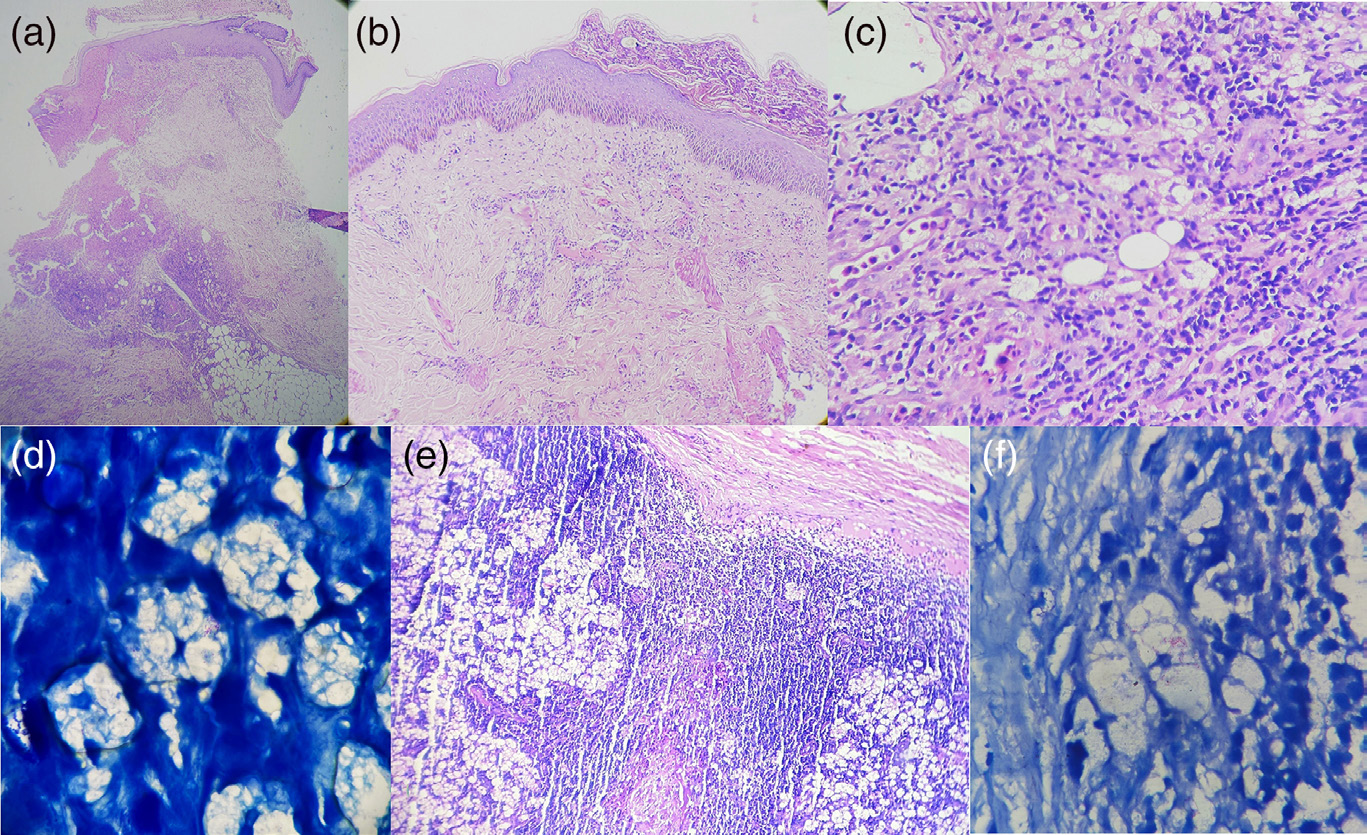
Findings of biopsy from margins of ulcers and lymph nodes. (a) Infiltrates following neuro-vascular bundles; haematoxylin and eosin (H&E) staining, 40×. (b) Fibrinoid degeneration of vessel walls; H&E staining, 100×. (c) Foamy histiocytes with admixed neutrophils; H&E staining, 400×. (d) Presence of solid and fragmented lepra bacilli; Fite-Faraco staining, 1000×. (e) Infiltration of foamy histiocytes in a lymph node; H&E staining, 100×. (f) Presence of lepra bacilli in a lymph node; Fite-Faraco staining, 1000×.

Punch biopsy of the skin from margins of ulcers demonstrated infiltrates of foamy histiocytes and lymphocytes following neurovascular bundles, admixed with neutrophils and signs of vasculitis (Fig. 2a–c). Fite-Faraco staining revealed fragmented and granular acid fast bacilli (Fig. 2d). Biopsy of inguinal lymph nodes showed infiltration of foamy histiocytes and presence of acid fast bacilli (Fig. 2e, f).

The presence of *M. leprae* in lymph node biopsies and *M. lepromatosis* in both lymph node and skin biopsies were confirmed by standard PCR (Table 1) [1, 2], followed by DNA sequencing of PCR amplicons.

Based on the above clinical and laboratory findings, a final diagnosis of DLL with LP due to dual infection with *M. leprae* and *M. lepromatosis* was established and the patient was started on ALT (rifampicin, minocycline, ofloxacin and clofazimine) with oral prednisolone (1 mg kg^–1^) and thalidomide (200 mg twice day). One month after starting this therapy, the patient did not show any improvement in skin lesions or systemic complaints. Laboratory markers of inflammation remained high. Given the presence of a severe neutrophilic response, adalimumab was administered subcutaneously (40 mg) as an adjuvant therapy after discussing and gaining consent for its off-label use with the patient. The patient reported mild improvements after the first dose of adalimumab but presented with increased skin lesions and systemic complaints (fever and joint pain) after the second dose (Fig. 1b). In view of the worsening clinical condition and persistent high laboratory markers of inflammation, off-label use of oral tofacitinib was discussed with the patient and consent for its use was given. One month after administration of tofacitinib the patient reported marked healing of the skin ulceration and cessation of new lesions (Fig. 1c). Laboratory evaluation revealed leukocytosis returning to a normal range and other inflammatory markers showing reducing trends. The patient was continued on ALT and oral steroids throughout this treatment period; oral thalidomide therapy was discontinued after commencement of tofacitinib. At the time of writing of this report, the patient is in remission of LP and on regular ALT.

### Discussion

*M. lepromatosis* has been recognized as causing DLL and severe LP from endemic countries in South America and Southeast Asia [3, 4]. Dual infection with *M. leprae* and *M. lepromatosis* has been confirmed in around 14 % cases where species identification was performed using genomic techniques [5]. Our patient had a clinical presentation of DLL and LP with dual infection from both species. These findings corroborate presentations from other endemic countries and represent the first confirmation of dual infection in India. Involvement of lymph nodes in patients with leprosy, particularly at the lepromatous pole, is well established. Our case is unique in its presentation where generalized lymphadenitis was the initial disease presentation. In the absence of characteristic infiltrated skin lesions associated with leprosy, it was misdiagnosed as tuberculous lymphadenitis. It is thus pertinent to keep this presentation in mind by clinicians as it can be confused with other causes of generalized lymphadenopathies and diagnosis may be delayed, particularly in patients with DLL. The LP involves a severe necrotizing skin reaction in cases of DLL with systemic manifestations and can be fatal if left untreated. Systemic GC along with ALT remains the mainstay of treatment for LP. For non-responding cases or patients with a persistent reaction, alternative therapies such as thalidomide and pentoxifylline have been utilized along with GC. Recently, biological therapy (e.g. TNF-α inhibitors) has been successfully used in the management of recalcitrant lepra reactions. [6] Our patient showed no improvement in skin lesions or systemic complaints despite use of GC, thalidomide and a TNF-α inhibitor. Studies on the pathogenesis of the type 2 lepra reaction show increase expression of Th2 cytokines such as IL4, IL5 and IL6 [7]. Recently, the role of Th17 cells has also been established in the pathogenesis of LP [8]. Tofacitinib is a JAK inhibitor that inhibits the intracellular signal transduction of pro-inflammatory cytokines. It is being increasingly utilized in various autoinflammatory conditions having a similar Th17 cytokine profile. With this rationale we decided to utilize it in our patient. The patient showed an excellent clinical response to tofacitinib as adjuvant to GC, which was further corroborated by improvement in laboratory markers of inflammation. Our case is unique as the first reported case of dual infection with *M. leprae* and *M. lepromatosis* from India, lymph node involvement as a presenting feature and excellent response to tofacitinib.

Limitations include that this is a report of a single case. Tofacitinib was used as adjuvant to GC and ALT, and thus not all clinical improvement may not be attributed to tofacitinib alone. Larger case series or randomized trials are needed before tofacitinib can be firmly established in the management of lepra reactions.

## References

[1] Donoghue HD, Holton J, Spigelman M (2001). PCR primers that can detect low levels of *Mycobacterium leprae* DNA. J Med Microbiol.

[2] Han XY, Quintanilla M (2015). Diffuse Lepromatous leprosy due to *Mycobacterium lepromatosis* in Quintana Roo, Mexico. J Clin Microbiol.

[3] Han XY, Seo Y-H, Sizer KC, Schoberle T, May GS (2008). A new *Mycobacterium* species causing diffuse lepromatous leprosy. Am J Clin Pathol.

[4] Widiatma RR, Sukanto H (2019). Diffuse lepromatous leprosy caused by dual infection of *Mycobacterium leprae* and *Mycobacterium lepromatosis*: a case report. Dermatol Reports.

[5] Han XY, Sizer KC, Velarde-Félix JS, Frias-Castro LO, Vargas-Ocampo F (2012). The leprosy agents *Mycobacterium lepromatosis* and *Mycobacterium leprae* in Mexico. Int J Dermatol.

[6] Cogen AL, Lebas E, De Barros B, Harnisch JP, Faber WR (2020). Biologics in leprosy: a systematic review and case report. Am J Trop Med Hyg.

[7] Yamamura M, Wang XH, Ohmen JD, Uyemura K, Rea TH (1992). Cytokine patterns of immunologically mediated tissue damage. J Immunol.

[8] Afzali B, Lombardi G, Lechler RI, Lord GM (2007). The role of T helper 17 (Th17) and regulatory T cells (Treg) in human organ transplantation and autoimmune disease. Clin Exp Immunol.

